# Anti-Inflammatory Effects of Recombinant Human PDCD5 (rhPDCD5) in a Rat Collagen-Induced Model of Arthritis

**DOI:** 10.1007/s10753-014-0008-x

**Published:** 2014-09-02

**Authors:** Juan Xiao, Ge Li, Jia Hu, Liujing Qu, Dalong Ma, Yingyu Chen

**Affiliations:** 1 Key Laboratory of Medical Immunology, Ministry of Health, Peking University Health Science Center, Beijing, 100191 China; 2 Peking University Center for Human Disease Genomics, Beijing, 100191 China

**Keywords:** PDCD5, regulatory T cells, collagen-induced arthritis, Th17, Th1

## Abstract

Programmed cell death 5 (PDCD5) was first identified as a gene upregulated in cells undergoing apoptosis. We recently demonstrated the inhibitory effect of PDCD5 on experimentally induced autoimmune encephalomyelitis. In this study, we investigated the anti-inflammatory effects of recombinant human PDCD5 (rhPDCD5) in a rat collagen-induced arthritis (CIA) model. We find that vaccination of collagen II (CII) induced CIA rats with rhPDCD5 significantly delayed the occurrence and reduced the severity of CIA rats. rhPDCD5 also restored the loss of Foxp3^+^ regulatory T (Treg) cells and decreased the population of Th1 and Th17 in CIA rats. Simultaneously, rhPDCD5 treatment suppressed the production of pro-inflammatory cytokines (interleukin (IL)-6, IL-17A, tumor necrosis factor-α (TNF-α), and interferon gamma (IFN-γ)) and increased the secretion of anti-inflammatory cytokines (transforming growth factor beta 1 (TGF-β1) and IL-10) in CIA rats. In addition, rhPDCD5 inhibited the ability of CII to induce proliferation of splenocytes and lymph node cells (LNCs) and promoted the CII-activated CD4^+^ cell apoptosis. These results of rhPDCD5-treated CIA rats were similar with those of recombinant human TNF-α receptor IgG Fc (rhTNFR:Fc). Thus, to our knowledge, we provide the first evidence that rhPDCD5 may be an efficient approach to diminishing exacerbated immune responses in CIA, indicating its therapeutic potential in the treatment of rheumatoid arthritis and other autoimmune diseases.

## INTRODUCTION

Rheumatoid arthritis (RA) is a chronic, systemic, inflammatory disease, characterized by inflammatory cell infiltration in the synovial tissue, synovial pannus formation, and it results in subsequent bone erosions, leading to joint destruction and progressive disability. Although the etiology and pathogenesis of RA remain unknown, many studies suggest that pathogenic T cells, such as Th1 and Th17 cells, are activated during the disease process and accumulated in the inflamed synovium, leading to perpetuation of inflammation with massive tissue destruction [1]. On the other hand, there is ample evidence that CD4^+^CD25^+^Foxp3^+^ regulatory T (Treg) cells also play a critical role in autoimmune diseases, including RA [2]. Their numbers and activation status are associated with RA activity in general. Thus, therapeutic strategies targeting RA should consider the upregulation of Treg cell differentiation, as well as the inhibition of pro-inflammatory cells and pro-inflammatory cytokines.

Programmed cell death 5 (PDCD5) was first identified as an apoptosis-promoting protein [3]. The decreased expression of PDCD5 has been detected in many human tumors, and restoration of PDCD5 with recombinant protein can significantly sensitize different cancer cells to chemotherapies [4–7]. Recombinant human PDCD5 (rhPDCD5) has been shown to enter a variety of cells by clathrin-independent endocytosis and exert biological activities [7]. Studies on molecular mechanism of PDCD5 have been proposed that PDCD5 interacts with the histone acetyltransferase Tip60 and the transcription factor p53 to promote apoptosis [8–10]. PDCD5 can also interact with other molecules such as NF-κB p65 [11] and CCT [12], in which participate in the regulation of cell apoptosis.

Clinical literature suggests that PDCD5 is involved in some autoimmune diseases and inflammatory processes [13–19]. In patients with RA, the levels of PDCD5 protein are inversely associated with the levels of pro-inflammatory cytokines interleukin (IL)-17 and tumor necrosis factor-α (TNF-α) [17, 18]. In patients with psoriasis, the downregulated level of PDCD5 mRNA is closely linked with its DNA methylation [19]. Recently, we have shown that PDCD5 interacts with Foxp3, increases acetylation of Foxp3 in synergy with Tip60 and enhances the repressive function of Foxp3 [20]. In *PDCD5* transgenic mice, overexpression of PDCD5 increased the level of Foxp3 protein and percentage of CD4^+^CD25^+^Foxp3^+^ cells, alleviating the severity of experimentally induced autoimmune encephalomyelitis (EAE) in *PDCD5tg* mice. The beneficial effect of PDCD5 resulted from increases of Treg cell frequency, reducing the predominant pathogenic Th17/Th1 response. This is the first report revealing that PDCD5 activity in T cells suppresses autoimmunity by modulating Foxp3-Tregs axis [20].

In view of the fact that rhPDCD5 can enter cells and exert biological activities [21], the present study was designed to assess the efficacy of rhPDCD5 in collagen-induced arthritis (CIA) rats. Data obtained from experiments indicate that rhPDCD5 has an anti-inflammatory effect in CIA rats.

## MATERIALS AND METHODS

### Reagents and Antibodies

Bovine type II collagen (CII) and complete Freund’s adjuvant (CFA) were purchased from Chondrex (Redmond, WA, USA). Anti-interferon gamma (IFN-γ) and anti-IL-17A antibodies were purchased from BD (San Diego, CA, USA). Foxp3 intracellular staining kit and cytokine detecting enzyme-linked immunosorbent assay (ELISA) kits were purchased from eBioscience (San Diego, CA, USA). Recombinant human tumor necrosis factor-α receptor IgG Fc fusion protein (rhTNFR:Fc) was obtained from Shanghai CP Guojian Pharmaceutical Co., Ltd (Shanghai, China). Phorbol 12-myristate13-acetate (PMA) and ionomycin were purchased from Sigma-Aldrich (St Louis, MO, USA) Recombinant human PDCD5 protein was supplied by Beijing Biosea Biotechnology Co., Ltd (Beijing, China). The endotoxin activity of the PDCD5 protein received was <10 EU/mg as detected using the limulus amebocyte lysate assay, and the purity of the rhPDCD5 protein was >95 %.

### Induction, Treatment, and Measurement of CIA in Rats

Female Wistar rats (150 to 200 g) were obtained from the Deparment of Laboratory Animal Science (Peking University Health Science Center, Beijing, China). They were kept under pathogen-free conditions and had free access to food and water during the experimental period. The experiments were conducted in a facility accredited by the Association for Assessment and Accreditation of Laboratory Animal Care (AAALAC) and conducted under Institutional Animal Care and Use Committee guidelines.

Experimental rats were randomly divided into four groups, including normal group, CIA + ovalbumin (OVA) group, CIA + rhTNFR:Fc group, and CIA + rhPDCD5 group. On day 0, all arthritic rats were intradermally injected with 0.5-mL CFA emulsion containing 200 μg CII at the back and base of the tail, followed by a booster injection 7 days later using the same method. The normal rats were injected with 0.5-mL sterilized physiological saline only. During the experiment, an optimal dose of rhPDCD5 (14 mg/kg) was injected intraperitoneally on alternate days from day 2 to day 26. As control, OVA (14 mg/kg) was injected in the same manner. RhTNFR:Fc (3.5 mg/kg, positive control) was injected every 3 days from day 2 to day 26. The CIA clinical score for each carpal tarsal joint of the rats was evaluated as follows: 0 = normal, 1 = slight swelling and erythema, 2 = pronounced edema, and 3 = joint rigidity. Scores for the four carpal tarsal joints measured each week were combined to give a global clinical score of a maximum of 12 for each rat.

### Tissue Preparation and Histomorphological Evaluation

For histological morphology analysis, rats were killed on day 28 after the first immunization and carpal tarsal joints of rats in each group were removed and fixed in 4 % paraformaldehyde (pH 7.4) for 25 h. Decalcification was completed in 10 % EDTA solution, and then the samples were embedded in paraffin. Thereafter, they were cut into 5-μm sagittal sections and stained with hematoxylin and eosin (H&E) and safranin O dye. Histopathological images of carpal tarsal joints were taken by light microscope. Histopathological assessments were coded for blind observation, and carpal tarsal joints were scored histopathologically using a qualitative scale from 0 to 4 based on the degree of inflammation, pannus formation, cartilage destruction, and bone erosion associated with arthritis. The scoring scale was defined as follows: 0, no lesion; 1, minimal inflammatory infiltration; 2, mild inflammatory infiltration and synovial hyperplasia; 3, pannus formation with cartilage degeneration; and 4, heavy inflammatory infiltration and debris in the joint, severe chondrocytes, and cartilage matrix loss with new bone tissue substitution, bone destruction.

### Flow Cytometry Analysis

The spleen and draining lymph nodes (LNs) from the different groups were harvested at 28 days after the first immunization to prepare single-cell suspensions. Peripheral blood mononuclear cells (PBMCs) were prepared using Ficoll-Paque separation media (Dakewei, Shenzhen, China). To quantify the number of Tregs, splenocytes, lymph node cells (LNCs), and PBMCs were surface-stained with anti-rat CD4-FITC/CD25-APC. After fixation at 4 °C for 30 min, cells were permeabilized and stained with anti-rat Foxp3-PE antibody. The stained cells were detected using the FACSCalibur flow cytometer (BD Biosciences, San Jose, CA, USA).

To quantify IFN-γ and IL-17 expressions in CD4^+^ T cell, cells were stimulated with PMA plus ionomycin in the presence of brefeldin A for 5 h, followed by surface staining with anti-rat CD4-FITC and permeabilization for staining with anti-rat IFN-γ-PE and IL-17-PE. The stained cells were detected by the FACSCalibur flow cytometer as previously described [20].

### Apoptosis Assay

The Annexin V-FITC Apoptosis Detection Kit (Beijing Biosea Biotechnology Co., Ltd., Beijing, China) was used according to the manufacturer’s instructions. Briefly, LNCs from different rats were harvested and stimulated with 20 μg/ml of CII. After 48 h, cells were collected and stained with anti-rat CD4-PE and AnnexinV-FITC, and percentages of CD4^+^Annexin V^+^ double positive cells were analyzed by the FACSCalibur flow cytometer.

### Detection of Cytokines

Serum and knee joint synovial fluids from different group were collected at 28 days after the first immunization, and concentrations of cytokines were measured using ELISA kits according to the manufacturer’s instructions. The withdrawal of synovial fluid was performed as follows: joint cavity was opened; the patellar ligament was carefully removed; and then 200 μl of 0.9 % NaCl was injected intraarticularly into each knee. Subsequently, the synovial fluid was aspirated with the same syringe.

For detecting cytokines in culture supernatants, splenocytes and LNCs collected from each group were plated (5 × 10^5^ cells/well) in 96-well plates and stimulated in triplicate wells with 20 μg/ml of CII. After 48 h, cytokine levels in the supernatants were detected by ELISA kits.

### Lymphocyte Proliferation Assay

Splenocytes and LNCs from different rats were seeded at 5 × 10^5^ cells/well in 96-well plates and stimulated with 20 μg/ml of CII for 48 h. Then, cells were pulsed with 1 μCi/well [^3^H] thymidine (MP Biomedicals, USA) and incubated for additional 8 h. The results were expressed as mean [^3^H] thymidine incorporation (cpm) ± standard deviation (SD).

### Statistical Analysis

The results were evaluated using ANOVA with subsequent comparisons by Student *t* test for paired or nonpaired data, as appropriate. Statistical significance was defined as *p* < 0.05.

## RESULTS

### rhPDCD5 Suppresses CIA Development and Protects Against Joint Destruction

To investigate the *in vivo* role of rhPDCD5 in the regulation of autoimmune diseases, we used a well-established CIA rat model which displayed many of the pathological features of human RA [22]. This arthritic rat model has been used extensively to analyze the anti-inflammatory effects of newly developed drugs on chronic arthritis.

**Fig. 1:**
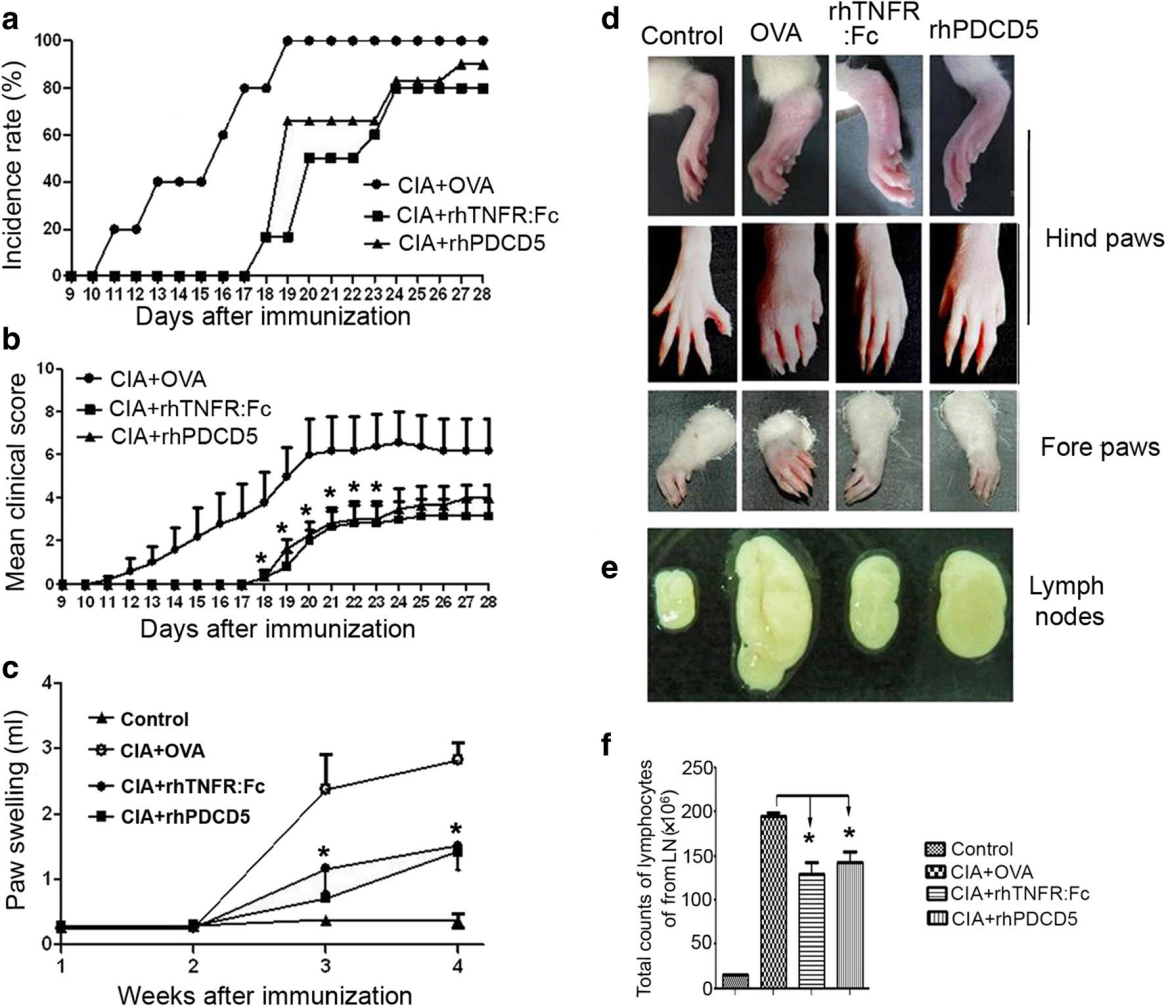
rhPDCD5 induces protective effects against CIA. **a** Incidence of CIA, defined as a total arthritis score ≥2 in each rat (*n* = 6). **b** Severity of arthritis during the course of CIA, as evaluated by the arthritis score (*P < 0.05 vs. CIA + OVA, *n* = 6). **c** Paw swelling was individually evaluated by the volumetric method described in the “MATERIALS AND METHODS” section (*P < 0.05 vs. CIA + OVA, *n* = 6). **d** Photographs of the hind paws and fore paws on day 28 after the first CII immunization. **e** Representative of inguinal LN from different groups. **f** Total counts of lymphocytes in inguinal LN from different groups (*n* = 6).

Arthritic rats were seen on day 11∼12 in OVA + CIA rats and reached a peak on day 20 after the induction of CIA (Fig. 1a, b and c). In contrast, CIA rats treated with either rhPDCD5 or rhTNFR:Fc showed a delayed onset of disease, as well as significant reductions in mean clinical score and paw swelling. Rats treated with either rhPDCD5 or rhTNFR:Fc showed less tumid of paws than the control group (Fig. 1d). OVA + CIA rats had markedly enlarged inguinal LN, which were much larger than either rhPDCD5- or rhTNFR:Fc-treated CIA rats (Fig. 1e). Total lymphocytes of inguinal LN were also counted, and rhPDCD5 + CIA rats had significantly fewer lymphocytes compared to OVA + CIA rats. Similar trend was observed in rhTNFR:Fc + CIA rats (Fig. 1f).

**Fig. 2:**
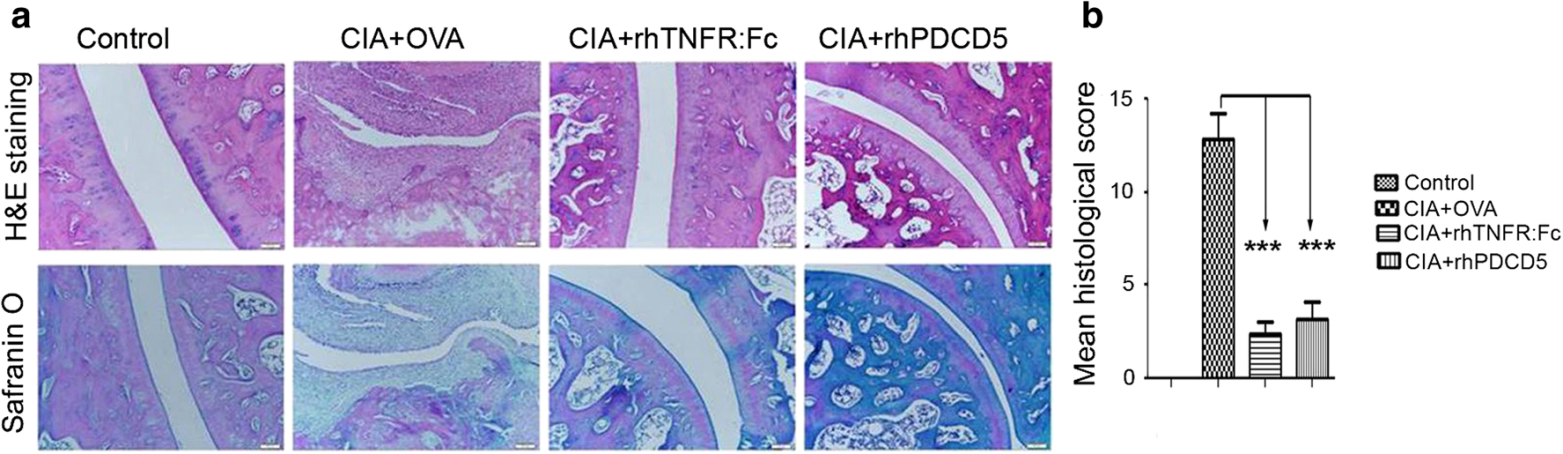
rhPDCD5 alleviates inflammatory histopathology of CIA. Histological examination of ankle joints. **a** Histology samples of carpal tarsal joints from four groups were stained with H&E and safranin O dye and observed with light microscope (original magnification ×100). Images are representative of *n* = 6 rats per group. **b** Histological scores of the four individual carpal tarsal joints were averaged for each rat, and mean scores for six rats selected per group are shown (***P < 0.0005).

The benefit of rhPDCD5 on CIA rats was further verified by histological examination. At day 28 after immunization, carpal tarsal joints of CIA rats were examined after H&E and safranin O staining. We observed that OVA + CIA rats developed inflammatory cell infiltration, pannus formation, cartilage destruction, and bone erosion with pannus replacing the articular cartilage overlying the bone. In contrast, the histological appearance of the carpal tarsal joints in rhPDCD5 + CIA rats demonstrated well-preserved joint space with minimal inflammatory exudates, normal structure of knee cartilage, and subchondral bone (Fig. 2a). Consistent with observations, the average histological score was markedly lower in rhPDCD5 + CIA rats compared with the OVA + CIA group, and similar trend was observed in rhTNFR:Fc + CIA rats (Fig. 2a, b).

### rhPDCD5 Increases Treg Frequency but Reduces Th1 and Th17 Response in CIA Rats

**Fig. 3:**
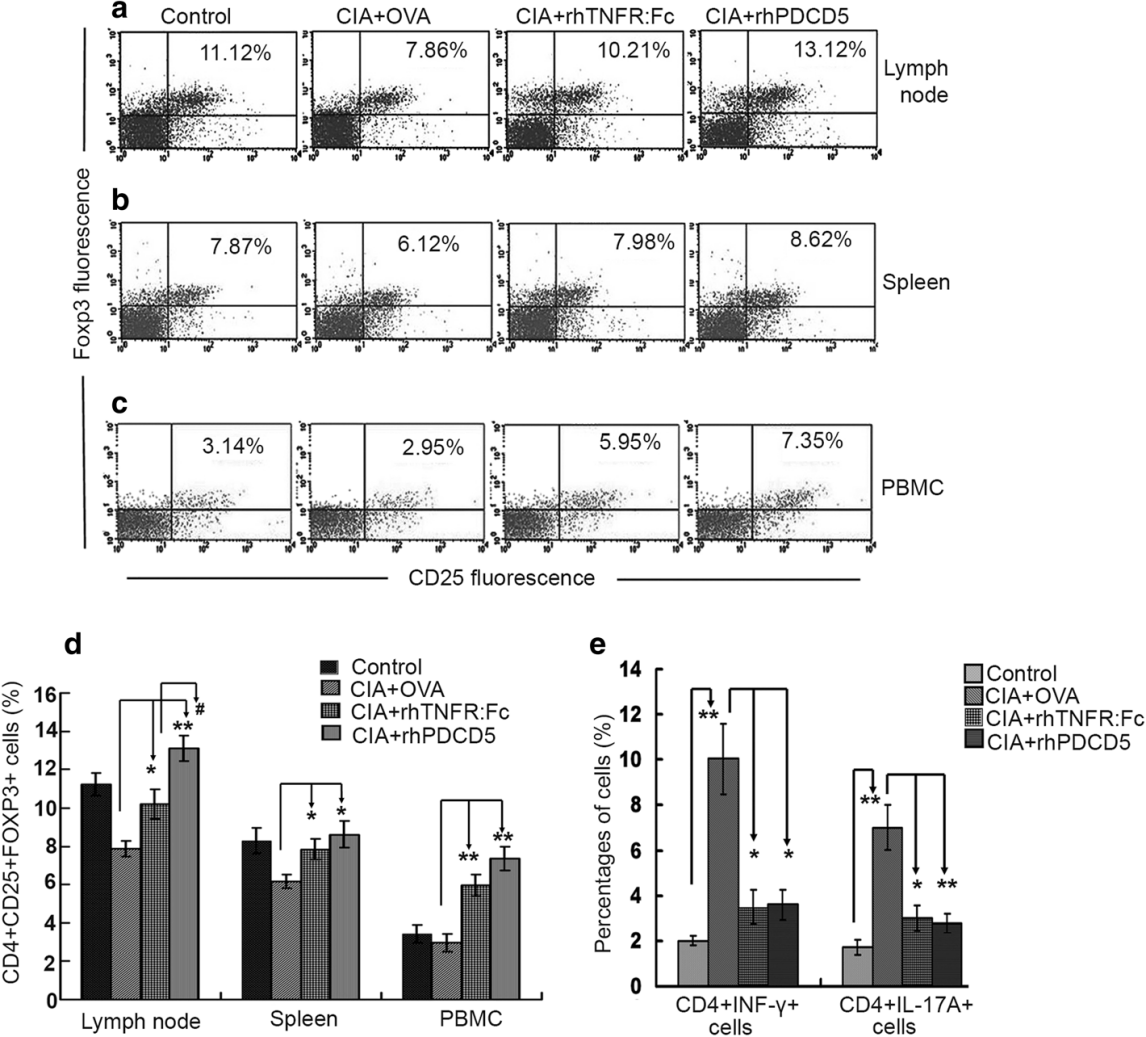
rhPDCD5 increases the percentages of Tregs and reduces the population of Th1 and Th17. Representative data of CD4^+^CD25^+^Foxp3^+^ cells analyzed by flow cytometry in draining lymph node (**a**), spleen (**b**), and PBMC (**c**). **d** Quantification of the proportion of CD4^+^CD25^+^Foxp3^+^ Tregs in lymph node, spleen, and PBMC. **e** Quantification of the percentage of CD4^+^IFN-γ^+^ and CD4^+^IL-17A^+^ cells in lymph node. Results are expressed as means ± SD (*P < 0.05, ** *P* < 0.005; #*P* < 0.05, *n* = 6).

Previous studies demonstrated that the percentages of Tregs are increased, and Th17/Th1 effectors are decreased in *PDCD5tg* mice with EAE [19]. We want to know whether rhPDCD5-treated CIA rats can also exhibit similar results. Therefore, we assessed the profiles of CD4^+^ T cell subsets in splenocytes, LNCs, and PBMCs from different CIA rats. As shown in Fig. 3a, b and c, the percentage of CD4^+^CD25^+^Foxp3^+^ Tregs was significantly increased in rhPDCD5 + CIA rats, compared with the OVA + CIA rats. Similar results were observed in the rhTNFR:Fc + CIA group. Interestingly, rhPDCD5 + CIA rats showed a higher population of Tregs in LN, compared with rhTNFR:Fc + CIA group (Fig. 3d). Simultaneously, the frequency of CD4^+^IFN-γ^+^ Th1 and CD4^+^IL-17A^+^ Th17 cells in rhPDCD5 + CIA and rhTNF:Fc + CIA rats was significantly decreased compared with OVA + CIA rats (Fig. 3e).

### rhPDCD5 Decreases the Production of Pro-Inflammatory Cytokines and Increases Anti-Inflammatory Cytokines in CIA Rats

**Fig. 4:**
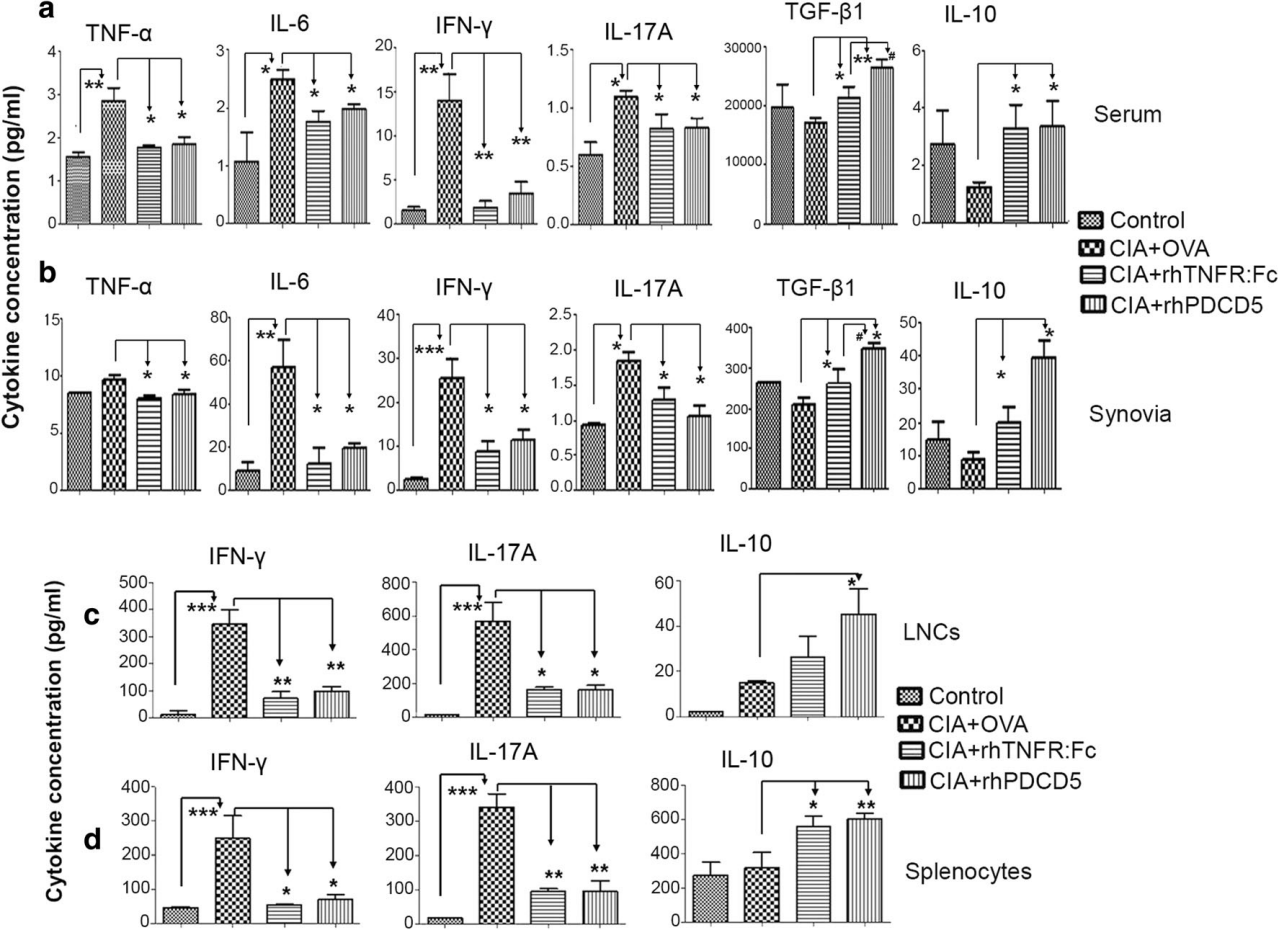
rhPDCD5 reduces the levels of pro-inflammatory cytokines in the serum, synovial, and culture supernatant. Serum (**a**) and synovia (**b**) were collected from the four groups on day 28, and cytokine concentrations were determined by ELISA. Data are means ± SD (*P < 0.05, ** *P* < 0.005, and ***P < 0.0005; #*p* < 0.05, *n* = 6). Draining LNCs (**c**) and splenocytes (**d**) were obtained from four groups and cultured with CII (20 μg/ml) for 48 h, and the cytokine concentrations in the culture supernatants were determined by ELISA. Data are means ± SD (*P < 0.05, **P < 0.005, and ***P < 0.0005, *n* = 6).

Treg cells suppress immune response through numerous mechanisms, including the production of anti-inflammatory cytokines and direct cell to cell contact. Therefore, we further examined the effect of rhPDCD5 on the levels of various pro-inflammatory and anti-inflammatory cytokines in CIA rats. As shown in Fig. 4a, b, rhPDCD5 significantly decreases the levels of IL-6, IL-17A, TNF-α, and IFN-γ, while increases the levels of TGF-β1 and IL-10 both in serum and synovial, compared with OVA + CIA rats. RhTNFR:Fc + CIA group showed similar effects to rhPDCD5. Moreover, rhPDCD5 + CIA rats showed higher levels of TGF-β1 both in serum and synovial than rhTNFR:Fc + CIA rats (Fig. 4a, b).

Further, we examined the cytokine production by draining LNCs and splenocytes *ex vivo*. Single-cell suspensions were cultured in the presence of bovine CII (20 μg/ml) for 48 h, and supernatants were analyzed by ELISA. It was clear that the production of IFN-γ and IL-17A were decreased in cultured LNCs and splenocytes from either rhTNFR:Fc- or rhPDCD5-treated CIA rats, compared to those OVA-treated group (Fig. 4c, d), whereas the levels of IL-10 were increased.

### rhPDCD5 Suppresses CII-Induced Cell Proliferation and Promotes Cell Apoptosis

**Fig. 5:**
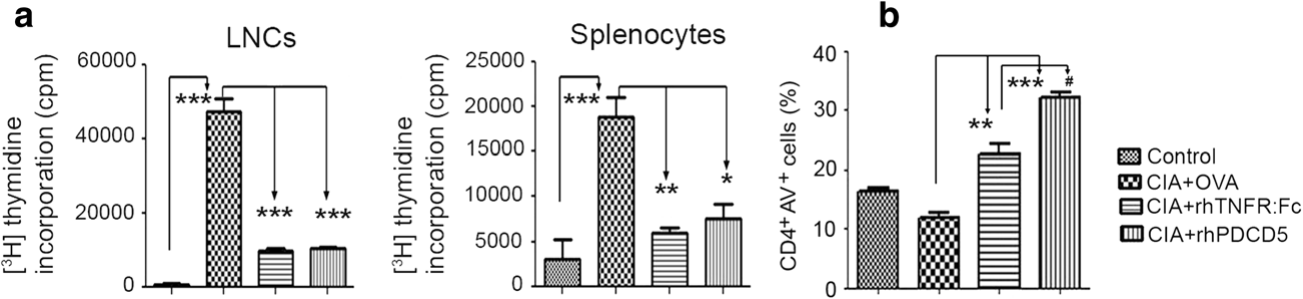
rhPDCD5 suppresses CII-induced cell proliferation and promotes cell apoptosis. **a** LNCs and splenocytes were obtained from the four groups on day 28 and cultured in the presence of CII (20 μg/ml) for 48 h. Cell proliferation was determined by the uptake of [^3^H] thymidine incorporation. Data are means ± SD (*P < 0.05, **P < 0.005, and ***P < 0.0005, *n* = 6). **b** LNCs were treated as in **a**, and cellular apoptosis was analyzed by FITC-Annexin V plus anti-CD4-PE staining and flow cytometry. Data are means ± SD (**P < 0.005 and ***P < 0.0005; #*p* < 0.05, *n* = 6).

To further determine whether rhPDCD5 could inhibit CII-specific lymphocyte responses, LNCs and splenocytes isolated from the different groups were stimulated *in vitro* with CII. Compared to the cells isolated from the control group, CII obviously stimulated the proliferation of both LNCs and splenocytes obtained from OVA + CIA rats. On the contrary, CII was less potent in stimulating the proliferation of LNCs and splenocytes isolated from either rhTNFR:Fc- or rhPDCD5-treated CIA rats (Fig. 5a). There was no significant difference between rhTNFR:FC and rhPDCD5 in terms of CII-specific lymphocyte responses.

Previous studies indicated that rhPDCD5 could promote cell apoptosis in variety of tumor cell [4–7]. We next investigated the effect of rhPDCD5 on activation-induced cell apoptosis in CII-stimulated CD4^+^ T cells. The treatment of LNCs was as same as cell proliferation assay. Treated LNCs were stained with anti-CD4-PE and FITC-Annexin V (AV) and analyzed by flow cytometry. We found that the percentage of CD4^+^AV^+^ cells were increased in either rhPDCD5- or rhTNFR:Fc-treated rats compared to that OVA + CIA rats (Fig. 5b). Importantly, the percentage of apoptosis from rhPDCD5 + CIA rats was larger than that of rhTNFR:Fc + CIA group.

## DISCUSSION

CIA model is commonly used to study human RA for its pathological, immunological, and clinical similarities. The fact that the inflammatory process in RA is chronic suggests that immune regulation in RA is disturbed. This disturbance is likely caused by an excessive inflammatory response together with a deficiency in the mechanisms that control the immune response.

We previous reported that *PDCD5tg* mice exhibited a systemic anti-inflammatory condition in EAE mice [20]. In the present study, we showed that rhPDCD5 possessed a similarly anti-inflammatory effect compared with rhTNFR:Fc, by diminishing the severity of arthritis and inhibiting the progression of CIA in rats. This strong efficacy may be associated with the increase of Treg cells and decrease prevalence of Th1 and Th17 cells. Simultaneously, treatment of rhPDCD5 caused the downregulation of pro-inflammatory cytokine productions and the upregulation of anti-inflammatory cytokines in the arthritic rats. These results indicate that the rhPDCD5 may be beneficial for the treatment of autoimmune arthritis. It should be noted that this study only partially explored the regulation of rhPDCD5 on cellular immunity. The influence of rhPDCD5 on humoral immunity will be further investigated.

Immunologically, Tregs comprise a subset of CD4^+^ lymphocytes that suppresses activation, proliferation, and effector responses of both innate and adaptive immune cells [23, 24]. Defects in Treg function have been demonstrated to lead to failed tolerance in human autoimmune diseases including RA [25]. Tregs can secrete TGF-β1 and IL-10 which suppress effecter T cell proliferation and cytokine production. It is thought that TGF-β1 can contribute to self-reactive T cell apoptosis and the maintenance of tolerance during lymphocyte maturation, thereby preventing the development of autoimmunity. The results of this study show that the administration of rhPDCD5 markedly increases the proportions of Tregs and the levels of TGF-β1 and IL-10 in CIA rats, which partly explain the immunosuppression effect of PDCD5 on the occurrence and development of CIA.

The Th17 subset is today recognized as a major player in synovial inflammation and bone erosion, mainly by the production of IL-17 [26, 27]. In fact, the pivotal role of Th17 in the pathogenesis of RA has been increasingly recognized as taking place via actions ascribed to IL-17. It is not clear whether Th17 cells consist of a specialized Th1 subtype or a different phenotype that would act in concert with Th1 cells. Several *in vivo* animal models and *in vitro* human studies have suggested that Th17 cells can be considered a decisive mediator of RA. The expression of IL-17 in the synovium and serum has been associated with the disease severity of RA [28]. Recently, two IL-17-neutralizing monoclonal antibodies were tested and appeared effective against RA in early stage clinical trials [29, 30]. Other studies have shown that Tregs can regulate the Th17 response in the RA patients. In our experiments, we observed that the increased Tregs stimulated by rhPDCD5 in CIA rats could downregulate the Th1/Th17 response and suppress the secretion of pro-inflammatory cytokines (IL-17A, IFN-γ, TNF-α, and IL-6). These results were similar with EAE model in *PDCD5tg* mice, indicating that rhPDCD5 may be an efficient approach to diminishing exacerbated immune responses in CIA.

Accumulating data proved that rhPDCD5 did not directly induce apoptosis but rather “primed” cells for markedly increased sensitivity to tumor cell apoptosis via different chemotherapeutics [4–7]. Here, we found that *in vitro* culture system, the proliferation levels of splenocytes, and LNCs isolated from rhPDCD5-treated rats were less potently induced by CII, which were required for the development of CIA model. Simultaneously, rhPDCD5-treated CD4^+^ cells stimulated by CII displayed the increased apoptosis sensitivity. Therefore, we postulate that rhPDCD5 treatment may prime self-reactive T cells to undergo increased activation-induced cell death in response to secondary exposure to self antigen and thus produce its therapeutic benefit by eliminating self-reactive T cells. In addition, previous report suggested that the levels of PDCD5 were reduced in RA synovial tissue (ST) and fibroblast-like synoviocytes (FLS) and the increased PDCD5 expression in RA patient-derived FLS undergoing apoptosis [16]. In a rat collagen-induced *in vivo* model of arthritis, we think that part of rhPDCD5 might enter FLS and increased the sensitivity of apoptosis which deserves further investigation. This may be one of the reasons of lower inflammation in rhPDCD5-treated CIA rats.

As a positive comparison to rhPDCD5 treatment, rats with CIA were treated with rhTNFR:Fc, an established therapy for experimental arthritis models and human RA [31]. In accordance with the results of previous experiments, rhTNFR:Fc has strong ability of suppressing progress in CIA. It has been shown that the Th1/Th17 cells in the periphery of RA patients are reduced, and Tregs are significantly increased after rhTNFR:Fc therapy [32]. In recent years, there have been many documents reported the multiple side effects accompanied with anti-TNF-α therapies, such as the increased risk of severe viral and bacterium infection [33, 34], anaphylaxis [35], and tumor [36]. Additionally, their considerable costs still limit their widespread utilization. The scientific community thus begins making an extraordinary effort to discover novel RA drugs that target crucial signals of activated cells or others. This present study together with previous research [20] implies that the immune-suppressing activity of rhPDCD5 may have therapeutic benefits for autoimmunity diseases.

In conclusion, our study provides the comprehensive assessment of immunosuppressive pathways of rhPDCD5 which induces a potent protective effect against CIA. Future studies will be required to determine whether this novel rhPDCD5-induced pathway can be exploited for therapeutic benefit.
